# Impact of dexamethasone on the incidence of ventilator-associated pneumonia and blood stream infections in COVID-19 patients requiring invasive mechanical ventilation: a multicenter retrospective study

**DOI:** 10.1186/s13613-021-00876-8

**Published:** 2021-05-31

**Authors:** Ines Gragueb-Chatti, Alexandre Lopez, Dany Hamidi, Christophe Guervilly, Anderson Loundou, Florence Daviet, Nadim Cassir, Laurent Papazian, Jean-Marie Forel, Marc Leone, Jean Dellamonica, Sami Hraiech

**Affiliations:** 1 Assistance Publique - Hôpitaux de Marseille, Hôpital Nord, Médecine Intensive Réanimation, 13015 Marseille, France; 2 Centre d’Études et de Recherches sur les Services de Santé et qualite de vie EA 3279, 13005 Marseille, France; 3 Service d’Anesthésie et de Réanimation, Aix Marseille Université, Assistance Publique Hôpitaux de Marseille, Marseille, France; 4 Service de Médecine Intensive Réanimation CHU de Nice et UR2CA, Université Cote d’Azur, Nice, France; 5 Institut Hospitalo-Universitaire Méditerranée Infection, Marseille, France; 6 Service de Médecine Intensive Réanimation, APHM, CHU Nord, 13015 Marseille, France

**Keywords:** COVID-19, Dexamethasone, Ventilator-associated pneumonia, Bloodstream infection, Mechanical ventilation

## Abstract

**Background:**

Dexamethasone decreases mortality in patients with severe coronavirus disease 2019 (COVID-19) and has become the standard of care during the second wave of pandemic. Dexamethasone is an immunosuppressive treatment potentially increasing the risk of secondary hospital acquired infections in critically ill patients. We conducted an observational retrospective study in three French intensive care units (ICUs) comparing the first and second waves of pandemic to investigate the role of dexamethasone in the occurrence of ventilator-associated pneumonia (VAP) and blood stream infections (BSI). Patients admitted from March to November 2020 with a documented COVID-19 and requiring mechanical ventilation (MV) for ≥ 48 h were included. The main study outcomes were the incidence of VAP and BSI according to the use of dexamethasone. Secondary outcomes were the ventilator-free days (VFD) at day-28 and day-60, ICU and hospital length of stay and mortality.

**Results:**

Among the 151 patients included, 84 received dexamethasone, all but one during the second wave. VAP occurred in 63% of patients treated with dexamethasone (DEXA+) and 57% in those not receiving dexamethasone (DEXA−) (*p* = 0.43). The cumulative incidence of VAP, considering death, duration of MV and late immunosuppression as competing factors was not different between groups (*p* = 0.59). A multivariate analysis did not identify dexamethasone as an independent risk factor for VAP occurrence. The occurrence of BSI was not different between groups (29 vs. 30%; *p* = 0.86). DEXA+ patients had more VFD at day-28 (9 (0–21) vs. 0 (0–11) days; *p* = 0.009) and a reduced ICU length of stay (20 (11–44) vs. 32 (17–46) days; *p* = 0.01). Mortality did not differ between groups.

**Conclusions:**

In this cohort of COVID-19 patients requiring invasive MV, dexamethasone was not associated with an increased incidence of VAP or BSI. Dexamethasone might not explain the high rates of VAP and BSI observed in critically ill COVID-19 patients.

**Supplementary Information:**

The online version contains supplementary material available at 10.1186/s13613-021-00876-8.

## Background

Among patients admitted to the intensive care unit (ICU) with a severe form of coronavirus disease 2019 (COVID-19), up to 80% [1] require invasive mechanical ventilation (MV). The mortality of these patients has been reported to be as high as 37% [1]. A large randomized controlled trial [2, 3] demonstrated that the use of dexamethasone resulted in a lower 28-day mortality in patients who were receiving invasive MV. Consequently, during the second wave of pandemic, dexamethasone has become the standard of care for COVID-19-related pneumonia in the ICU [4, 5]. The frequency of severe forms of acute respiratory distress syndrome (ARDS) [1, 6] exposes COVID-19 patients to a high risk of nosocomial infections [7]. Unexpected incidence of ventilator-associated pneumonia (VAP) has been recently reported in large series, as high as 50% for COVID-19 patients as compared to 30% for influenza [8]. Very high late-onset VAP rate has been described in patients under extracorporeal membrane oxygenation (ECMO) for severe COVID-19-related ARDS, again as compared with influenza patients [9]. Besides, a high frequency (67%) of bloodstream infections (BSI) [10] has been reported. Considering the wide use of dexamethasone during the second wave of COVID-19 pandemic, its role in the development of secondary bacterial infections comes under question. We therefore conducted a retrospective multicenter observational study, with a before–after design, to compare the incidence of VAP and BSI between patients of the first and second waves of COVID-19, before and after the publication of the RECOVERY [2] trial and the generalization of the use of dexamethasone. We also compared the duration of MV, length of stay and mortality according to the use of dexamethasone.

## Methods

### Study design and population

We conducted an observational retrospective study, with a before–after design, in three ICUs from two University hospitals in Southern France. Patients were included if they had been admitted to the ICU for a SARS-CoV-2 documented acute respiratory failure (from a pharyngeal or pulmonary sample RT-PCR) and required MV for at least 48 h. First wave of pandemic covered from March 10th to May 29th 2020 and second wave from August 14th to November 7th 2020 (end of the study period). Patients for whom withholding of treatments was decided during the first 48 h after ICU admission, aged under 18, deprived of liberty or without social protection were not included.

### Baseline assessment and data collection

Data were collected from the electronic patient’s file. Demographic characteristics, comorbidities, severity at ICU admission, date of COVID-19 first symptoms and RT-PCR positivity, date of ICU admission, date of intubation and invasive MV, need for ECMO, antiviral treatment, initial bacterial co-infection and antibiotics received at ICU admission, nosocomial infections (VAP and BSI) with microbiological documentation and recurrences, duration of invasive MV, ICU and hospital stay, status at day 28, day 90, ICU and hospital mortality were obtained. Occupational rates, invasive MV and ECMO, hydroalcoholic solution and antibiotic (piperacillin–tazobactam) consumption during the study period were recorded in the three participating ICUs. Patients were classified according to dexamethasone treatment, administered as in the RECOVERY trial (i.e. intravenous infusion of 6 mg/day during 10 days [2, 3]). Patients treated with dexamethasone were called “DEXA+”. In these patients, the delay from the initiation of dexamethasone to MV was recorded.

Patients who did not receive dexamethasone were called “DEXA−”.

In all patients, the use of rescue immunomodulatory therapies (RIT) was also recorded: the use of methylprednisolone for persistent ARDS as previously described [11], IL-1 inhibitors (anakinra), ruxolitinib or tocilizumab.

### VAP and BSI definitions

In patients receiving MV for at least 48 h, VAP was diagnosed when the following criteria were met [12–14]:

- New or progressive persistent infiltration on chest radiograph.
- At least two of the following:
  - New onset of fever.
  - Purulent endotracheal aspirate.
  - Leukocytosis or leucopenia.
  - Increased minute ventilation.
  - Arterial oxygenation decline.
  - Need for increased vasopressor infusion to maintain blood pressure.
  (In patients with ARDS, in which demonstration of radiologic deterioration is difficult, at least two of the preceding criteria sufficed).
- Positive culture from broncho-alveolar lavage (BAL) or a positive quantitative culture from endotracheal aspirate (ETA) specimen.

Patients with tracheostomy were considered at risk of VAP only during the period of MV.

ICU-acquired BSI was defined as at least one positive blood culture for bacteria or fungi, drawn at > 48 h after ICU admission was positive. For coagulase-negative staphylococci and other common skin contaminants, at least two consecutive blood cultures positive for the same pathogen at different times and sites were necessary [15].

### Relapse and recurrence of VAP

Recurrence of VAP was defined as a new onset of clinical symptoms following a partial or complete regression of the clinical signs after adequate antibiotic treatment with at least one positive bacterial culture at a significant concentration. Relapse was defined as a recurrence involving at least one of the initial causative bacteria; otherwise, it was considered a superinfection [7, 16].

### Study outcomes

The main study outcome was the incidence of VAP according to the use of dexamethasone. Crude and cumulative incidence, considering death, duration of MV (extubation) and rescue immunosuppressive therapy as competing factors were compared in DEXA+ and DEXA− groups.

Secondary outcomes were the incidence of BSI, recurrence of VAP or BSI, the time from MV to first VAP, the duration of MV, the ventilator-free days (VFD) at day 28 and day 60, ICU and hospital length of stay, ICU, hospital and day 60 mortalities according to the use of dexamethasone.

The same outcomes were analysed in the subgroups of patients who had received RIT associated or not with dexamethasone.

### Statistical analysis

Statistical analysis was performed using SPSS Version 20 (IBM SPSS Inc., Chicago, IL, USA) and cmprsk package from R software, version 3.2.3.

Continuous variables are expressed as means ± SD or as median with range (min, max), and categorical variables are reported as count and percentages. Comparisons of means values between two groups were performed using Student’s *t*-test or Mann–Whitney *U*. Comparisons of percentages were performed using Chi-square test or (Fisher’s exact test, as appropriate).

The cumulative incidence function with competing events was used to estimate VAP first episode [17]. Comparisons were done using the Fine and Gray model [18]. The competing risks for VAP were death, extubation and the use of rescue immunosuppressive therapy. All the tests were two-sided. Univariate and multivariate analyses were performed. The statistical significance was defined as *p* < 0.05. We secondary constructed four groups according the use of dexamethasone and rescue immunosuppressive therapy. Then, we performed for quantitative variables, analysis of the variance (ANOVA) with Bonferroni post hoc tests when significance was (*p* < 0.05) and for categorical variables, multiple comparisons with Kruskal–Wallis test with Tukey post hoc tests when significance was (*p* < 0.05).

## Results

### Patients’ characteristics at ICU admission

**Fig. 1:**
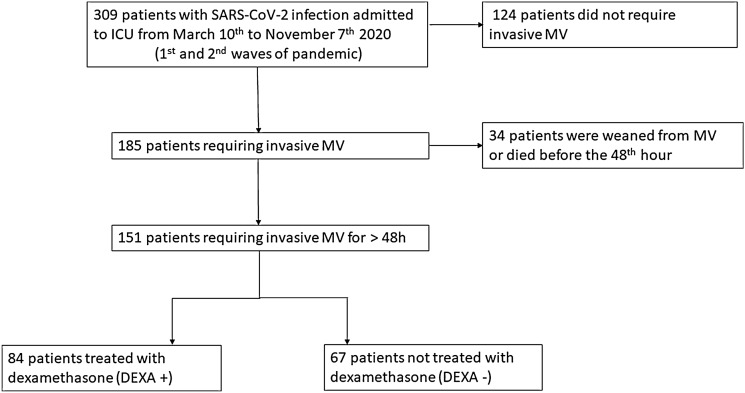
Study flowchart. *ICU* intensive care unit, *MV* mechanical ventilation

During the study period, 151 patients were included in the three participating ICUs (Fig. 1). Among them, 84 were treated with dexamethasone, all but one during the second wave of pandemic. Dexamethasone was administered before the period at risk of VAP or BSI. Patients’ characteristics at ICU admission are summarized in Table 1. There was no difference except for age, DEXA+ patients being slightly older (66 ± 11 vs. 62 ± 13 years old; *p* = 0.05), and antibiotics at ICU admission that were less frequently prescribed for DEXA+ patients (52 (62%) vs. 58 (87%); *p* = 0.001). Patients from the second wave were intubated later after ICU admission as compared to patients from the first wave (1 (0–4) day vs. 0 (0–1) day; *p* = 0.006). Bacterial co-infections were not different between groups. Dexamethasone was administered 1 (0–4) days before the beginning of MV.

**Table 1:** Patients main characteristics at ICU admission

	Overall (*n* = 151)	DEXA+ (*n* = 84)	DEXA− (*n* = 67)
Male, *n* (%)	120 (79)	65 (77)	55 (82)
Age, years ± SD	64 ± 12	66 ± 11	62 ± 13
SAPS 2, mean ± SD	42 ± 14	42 ± 13	42 ± 15
SOFA, mean ± SD	6 ± 4	5 ± 4	6 ± 3
Comorbidities, *n* (%)			
Hypertension	73 (48)	43 (51)	30 (45)
Diabetes mellitus	55 (36)	34 (40)	21 (31)
Obesity	44 (29)	24 (29)	20 (30)
Smoker	37 (25)	18 (21)	19 (28)
Chronic heart failure	27 (18)	12 (14)	15 (22)
Chronic respiratory failure	20 (13)	11 (13)	9 (13)
History of neoplasm	18 (12)	12(14)	6 (9)
Immunosuppression	11 (7)	6 (7)	5 (7)
Chronic renal failure	9 (6)	6 (7)	3 (4)
Antiviral agents, *n* (%)	22 (15)	0	22 (33)
Lopinavir–ritonavir	21 (14)	0	21 (31)
Remdesivir	1 (1)	0	1 (2)
Hydroxychloroquine, *n* (%)	37 (25)	2 (2)	35 (52)
Antimicrobial treatment at ICU admission, *n* (%)	110 (73)	52 (62)	58 (87)
Documented co-infection, *n* (%)	16 (11)	6 (7)	9 (13)
Time from hospital admission to ICU admission, days, median (IQR)	0 (0–2)	0 (0–2)	0 (0–2)
Invasive MV at ICU admission, *n* (%)	70 (46)	30 (36)	40 (60)
Time to intubation, days, median (IQR)	1 (0–2)	1 (0–4)	0 (0–1)
Time from dexamethasone to intubation, days, median (IQR)	–	1 (0–4)	–
ECMO, *n* (%)	29 (19)	15 (18)	14 (21)
Hydrocortisone for septic shock, *n* (%)	27 (18)	11 (13)	16 (24)

Data are presented as median and interquartile range or mean ± standard deviation or absolute value and percentage

### Incidence of VAP and BSI (Table 2)

**Table 2:** Patients outcomes according to treatment with dexamethasone

	Overall (*n* = 151)	DEXA+ (*n* = 84)	DEXA− (*n* = 67)	*p* value
At least 1 VAP and/or 1 BSI, *n* (%)	100 (66)	56 (67)	44 (66)	0.59
At least 1 VAP, *n* (%)	91 (60)	53 (63)	38 (57)	0.43
Second VAP episode, *n* (%)	34 (23)	22 (26)	12 (18)	0.23
Third VAP episode, *n* (%)	15 (10)	11 (13)	4 (6)	0.15
At least 1 BSI, *n* (%)	44 (29)	24 (29)	20 (30)	0.86
Second BSI episode, *n* (%)	14 (9)	7 (8)	7 (10)	0.66
Third BSI episode, *n* (%)	3 (2)	1 (1)	2 (3)	0.59
Invasive MV duration before first VAP, days, median (IQR)	6 (3–12)	5 (3–10)	9 (4–15)	**0.02**
Mortality at D28, *n* (%)	25 (17)	14 (17)	11 (16)	0.94
Mortality at D60, *n* (%)	39 (26)	23 (28)	16 (24)	0.57
Hospital mortality, *n* (%)	46 (32)	28 (35)	18 (27)	0.27
VFD D28, median (IQR)	0 (0–18)	9 (0–21)	0 (0–11)	**0.009**
VFD D60, median (IQR)	27 (0–49)	37(0–53)	25 (0–43)	0.12
Duration of mechanical ventilation, days, median (IQR)	17 (9–37)	14 (7–39)	24 (12–36)	**0.008**
ICU length of stay, days, median (IQR)	24 (15–45)	20 (11–44)	32 (17–46)	**0.01**
Hospital length of stay, days, median (IQR)	31 (20–49)	28 (18–47)	33 (24–53)	0.06

*p* values in bold were considered statistically significant

Data are presented as median and interquartile range or absolute value and percentage

VAP occurred, respectively, in 63 vs. 57% (*p* = 0.43) for DEXA+ and DEXA− patients. The incidence of VAP was 26‰ days under MV overall, 31‰ for DEXA+ patients and 21‰ for DEXA− patients (*p* = 0.16).The first VAP occurred earlier after the onset of invasive MV in the DEXA+ patients (5 (3–10) vs. 9 (4–15) days; *p* = 0.02).

**Fig. 2:**
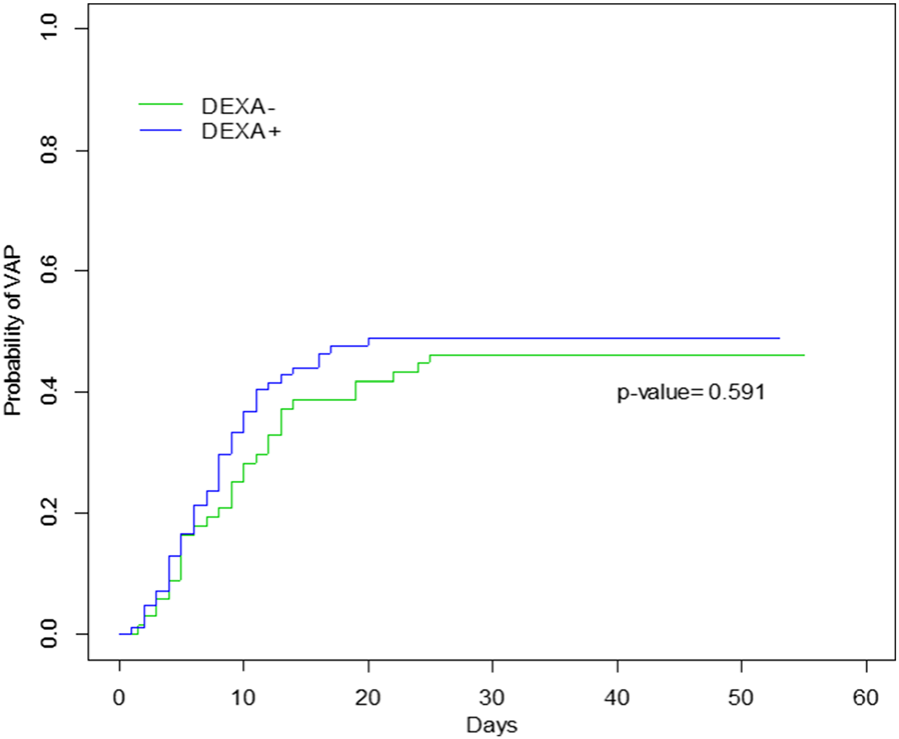
Estimated cumulative incidence of the first episode of ventilator-associated pneumonia (VAP) according to dexamethasone treatment, taking into account death, extubation and the use of rescue immunosuppressive therapy (RIT) as competing events. *p* values for differences between dexamethasone and no dexamethasone patients were 0.59 for VAP, 0.3 for death 0.94 for extubation and 0.61 for RIT. DEXA+: patients treated with dexamethasone, DEXA−: patients not treated with dexamethasone, VAP: ventilator-associated pneumonia

Duration of MV being different between groups, the cumulative incidence of VAP, considering death, duration of MV (extubation) and the use of late immunosuppression as competing factors was calculated and was similar in DEXA+ and DEXA− patients (*p* = 0.59) (Fig. 2). In a competing risks regression including age, main comorbidities, SOFA score, the use of antibiotics at ICU admission, the time from ICU admission to intubation, and the duration of ICU stay, dexamethasone was not an independent risk factor of VAP occurrence (Table 3).

**Table 3:** Multivariate analysis of the factors associated with the first occurrence of VAP considering death, extubation and RIT as competing factors

	CRH	95% CI	*p* value
Age	1.00	0.98–1.03	0.7
Comorbidities (0, 1, 2 or 3)	0.78	0.48–1.29	0.34
Dexamethasone use	1.07	0.65–1.77	0.79
SOFA score	0.98	0.99–1.02	0.48
Antibiotics at ICU admission	0.74	0.39	1.41
Time to intubation (days)	0.92	0.75–1.12	0.41
Duration of ICU stay (days)	1.01	1.02–1.08	0.09

*CRH* competing risk hazard, *CI* confidence interval

BSI occurred in 29% of patients in the DEXA+ group as compared with 30% for the DEXA− group (*p* = 0.86). The incidence of BSI was 10‰ days of ICU stay overall, 11‰ for DEXA+ patients and 9‰ for DEXA− patients (*p* = 0.6).

One hundred (66%) patients developed at least 1 VAP or 1 BSI during their ICU stay (56 (67%) in the DEXA+ group and 44 (66%) in the DEXA− group; *p* = 0.59).

Fifty-seven patients developed 1 VAP (31 DEXA+ and 26 DEXA−), 19 patients had 2 VAP (11 DEXA+ and 8 DEXA−) and 15 patients had 3 VAP (11 DEXA+ and 4 DEXA−).

Thirty patients developed 1 BSI (17 DEXA+ and 13 DEXA−), 11 patients had 2 BSI (6 DEXA+ and 5 DEXA−) and 3 patients had 3 BSI (1 DEXA+ and 2 DEXA−).

### Microbiology of VAP and BSI

Table 4 summarizes the microbiological details of first VAP in both groups.

**Table 4:** Microorganisms responsible for the first episode of VAP according to dexamethasone treatment

Pathogens responsible for 1st VAP	Overall (*n* = 127)	DEXA+ (*n* = 74)	DEXA− (*n* = 53)
Gram-negative pathogens, *n* (%)	84 (66)	53 (72)	31 (58)
Enterobacteriaceae	54 (64)	38 (72)	16 (52)
* Klebsiella aerogenes*	12 (22)	8 (21)	4 (25)
* Klebsiella pneumoniae*	11 (20)	9 (24)	2 (12)
* Klebsiella variicola*	2 (4)	1 (3)	1 (6)
* Klebsiella oxytoca*	2 (4)	2 (5)	0
* Morganella morganii*	5 (9)	3 (8)	2 (12)
* Hafnia alvei*	4 (7)	3 (8)	1 (6)
* Enterobacter cloacae*	7 (13)	6 (16)	1 (6)
* Proteus* spp.	3 (6)	2 (5)	1 (6)
* Serratia marcescens*	3 (6)	1 (3)	3 (19)
* Citrobacter*	2 (4)	2 (5)	0
* Escherichia coli*	3 (6)	1 (3)	2 (12)
Non-fermenting GNB, *n* (%)	27 (32)	12 (23)	15 (48)
* Pseudomonas aeruginosa*	22 (81)	8 (67)	14 (78)
* Stenotrophomonas maltophilia*	3 (11)	2 (17)	1 (7)
* Acinetobacter* spp.	2 (7)	2 (17)	0
* Haemophilus influenzae*	3 (4)	3 (6)	0
Gram-positive pathogens, *n* (%)	43 (34)	21 (28)	22 (42)
* Staphylococcus aureus*	28 (65)	15 (71)	13 (59)
Methicillin susceptible	25 (58)	12 (57)	13 (59)
Methicillin resistant	3 (7)	3 (14)	0
* Enterococcus* spp.	8 (19)	3 (14)	5 (23)
* Corynebacterium*	2 (5)	1 (5)	1 (5)

Data are presented as absolute value and percentage

Gram-negative bacteria (66%) and especially Enterobacteriaceae and non-fermenting Gram-negative bacilli were the most commonly retrieved pathogens during the first episode of VAP. Gram-positive pathogens were mainly methicillin-susceptible *Staphylococcus aureus* (MSSA) and *Enterococcus *spp. There was a trend towards more Enterobacteriaceae VAP in the DEXA+ group (72 vs. 52%; *p* = 0.06). Non-fermenting Gram-negative bacilli were more frequent in DEXA− patients (48 vs. 23%; *p* = 0.02).

Overall, 28 (31%) first VAP episode were polymicrobial with no difference between groups (*p* = 0.89).

BSI involving Gram-positive pathogens and especially *Enterococcus *spp*.* and coagulase-negative *Staphylococci* were the most frequent. However, Gram-negative bacteraemia was more frequent among DEXA+ patients (11 (39%) vs. 3 (13%); *p* = 0.04). The source of BSI was a bacteraemic pneumonia for 12 (27%) patients, a catheter-related BSI for 7 (16%) patients and was of unknown origin (primary BSI) for 25 (57%) patients. Microbiological details of BSI in the two groups are provided in Table 5.

**Table 5:** Microorganisms responsible for the first episode of BSI according to dexamethasone treatment

Pathogens responsible for 1st BSI	Overall (n = 51)	DEXA+ (*n* = 28)	DEXA− (*n* = 23)
Gram-negative pathogens, *n* (%)	14 (27)	11 (39)	3 (13)
Enterobacteriaceae	10	7	3
* Klebsiella variicola*	1	0	1
* Klebsiella aerogenes*	2	2	0
* Hafnia alvei*	1	1	0
* Enterobacter cloacae*	1	1	0
* Proteus* spp.	1	1	0
* Serratia marcescens*	2	1	1
* Escherichia coli*	2	1	1
Non-fermenting GNB, *n* (%)	4	4	0
* Pseudomonas aeruginosa*	3	3	0
* Acinetobacter* spp.	1	1	0
Gram-positive pathogens, *n* (%)	35 (69)	17 (61)	18 (78)
* Staphylococcus aureus*	7	3	4
Methicillin susceptible	6	3	3
Methicillin resistant	1	0	1
* Enterococcus* spp.	13	7	6
* Coagulase negative Staphylococci*	12	5	7
* Streptococci*	3	2	1
Moulds	2 (4)	0	2 (9)

Data are presented as absolute value and percentage

### VAP recurrences, relapses and superinfections

VAP recurrence was documented in 34 (37%) patients. The same pathogen was responsible for recurrence in 23 (68%) of them. The median time before recurrence was 12 (9–16) days. Recurrent VAP occurred in 22 (42%) vs. 12 (32%) DEXA+ and DEXA− patients, respectively. Microorganisms responsible for VAP recurrences are listed in Additional file 1: Table S1. Enterobacteriaceae and *Pseudomonas aeruginosa* were responsible for most VAP relapses.

### Clinical outcomes

Day 28, day 60 and hospital mortality did not differ according to dexamethasone treatment. However, DEXA+ patients had a shorter duration of MV (14 (7–39) vs. 24 (12–36) days; *p* = 0.008) and more VFD at day 28 (9 (0–21) vs. 0 (0–11) days; *p* = 0.009). They also had a reduced ICU length of stay (20 (11–44) vs. 32 (17–46) days; *p* = 0.01) (Table 2).

**Fig. 3:**
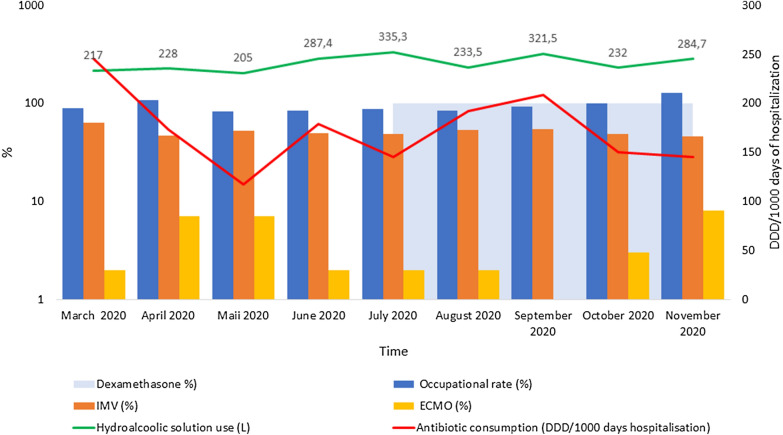
Constructing timelines showing the occupational rate, invasive MV and ECMO rates, hydroalcoholic solution consumption, antibiotic (piperacillin–tazobactam) consumption and dexamethasone across the study period. *DDD* daily defined dose, *ECMO* extracorporeal membrane oxygenation, *IMV* invasive mechanical ventilation

Figure 3 summarizes data on occupational rate, invasive MV and ECMO rates, hydroalcoholic solution consumption, monthly antibiotic (piperacillin–tazobactam) consumption and dexamethasone use.

### Use of rescue immunosuppressive therapy

RIT was used in 46 (30%) patients. There was no difference between the two groups regarding the use of at least one rescue therapy. The details of treatments are presented in Additional file 2: Table S2. The outcomes according to the use of RIT in both groups (DEXA+ and DEXA− patients) are provided in Additional file 3: Table S3. The use of RIT was associated with a higher rate of VAP and BSI regardless the use of dexamethasone.

## Discussion

In this retrospective cohort comparing the first and second waves of COVID-19 pandemic in three ICUs, dexamethasone was not associated with an increased incidence of VAP and BSI among patients under MV. Previous series had found discordant data on the frequency of nosocomial infections in COVID-19 patients [19, 20]. In our series, the incidence of VAP was higher than described in non-COVID ARDS [21] and comparable to the incidence reported in a recently published large COVID-19 cohort [6]. Noteworthy, in this latter study, very few patients received dexamethasone. The only study that reported the incidence of nosocomial infections in COVID-19 patients treated with dexamethasone found a 12% incidence of VAP and 8% BSI [19], however with a 28-day follow-up only. We performed a 60-day follow-up which might explain the higher incidence described herein. In our cohort, the incidence of BSI was higher than reported in non-COVID patients series [22, 23]. Our results are consistent with those from Buetti et al. [24] who reported 14.9% of BSI in the COVID-19 group and only 3.4% in the non-COVID-19 patients. In this study as in ours, corticosteroids did not increase the risk of developing BSI. The high rate of BSI we report might be due to the long duration of MV and ICU stay, partly explained by the 19% patients under ECMO. Surge in the number of critically ill patients challenging the full respect of usual infection control practices as well as the systematic use of gloves [25] have also been raised to explain the high rate of bacteremia. The disruption of the gut barrier caused by SARS-CoV-2 might also increase intestinal permeability and bacterial translocation, favouring the development of BSI, as it has been shown that SARS-CoV-2 productively infects human gut enterocytes [26]. In our cohort, Gram-negative pathogens and especially Enterobacteriaceae and *Pseudomonas aeruginosa* were predominant, as it has been shown in other series, but MSSA and *Enterococcus *spp. VAP were more frequently retrieved than in other series [6, 19].

Apart from dexamethasone treatment, several aspects of COVID-19 might lead to increased risks of nosocomial infections: the complexity of host-response to SARS-CoV-2 infection, including moderate to severe systemic inflammation and/or marked systemic immune suppression [27] as well as the pulmonary vasculopathy with endothelial dysfunction and endothelialitis [28, 29]. The high rate of relapses observed, especially in patients receiving adequate antibiotics with monitored pharmacokinetics [7], questions the local diffusion of antibiotics into “COVID-19” lungs.

Although not affecting the incidence of VAP or BSI, treatment with dexamethasone resulted in some differences between patients: VAP occurred earlier and involved less frequently non-fermenting Gram-negative bacteria but rather Enterobacteriaceae. This was unexpected as dexamethasone has been shown to increase susceptibility to *Pseudomonas aeruginosa* pneumonia in animal models through suppressing iNOS gene expression and peroxynitrite production [30]. The lower use of antibiotics (especially 3rd generation cephalosporins) received at the time of ICU admission during the second wave in dexamethasone patients has probably contributed to modify the patients microbiota. Indeed, guidelines published updated between the 2 first waves discouraged the systematic use of antibiotics given the relatively low rates of bacterial co-infections [4].

Patients receiving dexamethasone had more VFD at D28 as it was found in the CODEX-study [19], a shorter duration of MV and ICU length of stay. These results were in accordance with those from Villar et al. in non-COVID-19 ARDS patients [31]. However, in our cohort, mortality was not different between groups contrary to what was found in the RECOVERY-trial [3]. In a recently published study, Contou et al. [32] did not find any difference concerning the prognosis during the first and second waves. There was no difference in duration of MV, conversely to our results. However, in this paper, authors reported a high ICU mortality, especially for patients requiring MV (57% in the first wave and 75% during the second wave). In our cohort, we found a 38% hospital mortality. This mortality discrepancy might be related to different case mix and hardens comparison with our results.

Finally, we found an association between the use of rescue immunosuppressive treatment and a higher rate of VAP and BSI. Although of importance, this result deserves to be confirmed in a larger cohort and considering potential confounding factors.

Our study has several limitations that should be underlined. Firstly, it is a retrospective design. Secondly, given the relatively small size of the cohort, the study might be underpowered.

Third, the large use of antibiotics at ICU admission during the first wave of COVID-19 pandemic, very limited during the second wave, might have affected the microbiology of secondary bacterial complications. Dexamethasone patients were the less exposed to early antibiotics which might have affected the delay of VAP occurrence but not the global incidence. Fourth, the pulmonary samples used to diagnose VAP as well as the antibiotics prescribed differed according to physicians’ practices. However, we did not observe any difference in VAP or BSI incidence or recurrences between centres. Lastly, some patients received rescue immunomodulatory therapies later during their ICU stays [33], which might have favoured nosocomial infections. These patients were equally split in both groups and this confounding factor was considered as a competing event and did not modify the cumulative incidence of VAP.

## Conclusions

In this cohort of severe forms of COVID-19 patients requiring invasive MV, dexamethasone was not associated with an increase in the incidence of VAP or BSI. Dexamethasone treatment was associated with more ventilator-free days at day-28. Dexamethasone treatment might not explain the high rate of VAP and BSI observed in critically ill COVID-19 patients.

## Supplementary Information

**Additional file 1: Table S1.** Characteristics of recurrent VAP episodes according to dexamethasone treatment.

**Additional file 2: Table S2.** Use of rescue immunosuppressive therapy.

**Additional file 3: Table S3.** Patients outcomes according to treatment with dexamethasone and rescue immunosuppressive therapy.

## Data Availability

The datasets used and/or analysed during the current study are available from the corresponding author on reasonable request.

## Abbreviations

ARDS: Acute respiratory distress syndrome
BAL: Broncho-alveolar lavage
BSI: Blood stream infections
ECMO: Extracorporeal membrane oxygenation
ETA: Endotracheal aspirate
IL-1: Interleukin 1
ICU: Intensive care unit
MV: Mechanical ventilation
RT-PCR: Reverse transcription polymerase chain reaction
RIT: Rescue immunomodulatory therapies
SAPS II: Simplified Acute Physiologic Score II
SOFA: Sequential Organ Failure Assessment
VAP: Ventilator-associated pneumonia
VFD: Ventilator-free days
