# Supplementary material for: Impact of dexamethasone on the incidence of ventilator-associated pneumonia and blood stream infections in COVID-19 patients requiring invasive mechanical ventilation: a multicenter retrospective study
Source: Ann Intensive Care. 2021 May 31;11:87. doi: 10.1186/s13613-021-00876-8 (PMC8165680; doi:10.1186/s13613-021-00876-8)
Supplement: Supplementary file 1 — Additional file 1: Table S1. Characteristics of recurrent VAP episodes according to dexamethasone treatment. [file 13613_2021_876_MOESM1_ESM.docx]

| Characteristics | VAP 2 | | | VAP 3 | | |
| --- | --- | --- | --- | --- | --- | --- |
|  | **Overall (n=34)** | **DEXA + (n=22)** | **DEXA - (n=12)** | **Overall (n=15)** | **DEXA + (n=11)** | **DEXA - (n=4)** |
| Relapse, n (%) | 23 (68) | 16 (73) | 7 (58) | 10 (67) | 8 (72) | 2 (50) |
| Superinfection, n (%) | 11 (32) | 6 (27) | 5 (42) | 5 (33) | 3 (27) | 2 (50) |
| Days between VAP 1 and recurrence, median (IQR) | 12 (9-16) | 13 (10-17) | 10 (6-14) | 20 (17-29) | 21 (18-29) | 18 (15-26) |
| Pathogen responsible of recurrence, n (%) |  |  |  |  |  |  |
| *Enterobacteroaceae* | 14 (39) | 10 (43) | 4 (31) | 3 (20) | 3 (27) | 0 |
| *Pseudomonas aeruginosa* | 12 (35) | 6 (27) | 6 (50) | 5 (33) | 3 (27) | 2 (50) |
| *Achromobacter* spp. | 1 (3) | 1 (5) | 0 | 0 | 0 | 0 |
| *Acinetobacter* baumanii | 1 (3) | 1 (5) | 0 | 1 (7) | 1 (9) | 0 |
| *Enterococcus faecalis* | 1 (3) | 0 | 1 (8) | 0 | 0 | 0 |
| *Staphylococcus aureus* | 3 (9) | 2 (9) | 1 (8) | 1 (7) | 0 | 1 (25) |
| *Streptococcus* spp*.* | 2 (6) | 1 (5) | 1 (8) | 0 | 0 | 0 |
| Other | 1 (3) | 0 | 1 (8) | 1 (7) | 1 (9) | 0 |
| Polymicrobial | 2 (6) | 1 (5) | 1 (8) | 4 (27) | 3 (27) | 1 (25) |

**Table S1. Characteristics of recurrent VAP episodes according to dexamethasone treatment**

Data are presented as absolute value and percentage.
