# Supplementary material for: Impact of dexamethasone on the incidence of ventilator-associated pneumonia and blood stream infections in COVID-19 patients requiring invasive mechanical ventilation: a multicenter retrospective study
Source: Ann Intensive Care. 2021 May 31;11:87. doi: 10.1186/s13613-021-00876-8 (PMC8165680; doi:10.1186/s13613-021-00876-8)
Supplement: Supplementary file 2 — Additional file 2: Table S2. Use of rescue immunosuppressive therapy. [file 13613_2021_876_MOESM2_ESM.docx]

|  | Overall  (n=151) | DEXA +  (n=84) | DEXA –  (n=67) | P value |
| --- | --- | --- | --- | --- |
| Received rescue immunosuppressive therapy (at least one), n (%)  Late steroids (methylprednisolone), n (%)  Anti IL-1, n (%)  Ruxolitinib, n (%)  Tocilizumab, n (%) | 47 (31)  29 (19)  12 (8)  17 (11)  9 (6) | 27 (32)  15 (18)  3 (4)  8 (10)  8 (10) | 20 (30)  14 (21)  9 (13)  9 (13)  1 (1) | 0.76  0.64  0.03  0.45  0.04 |
|  |  |  |  |  |

Table S2. Use of rescue immunosuppressive therapy

IL-1: interleukin 1
