# Supplementary material for: Impact of dexamethasone on the incidence of ventilator-associated pneumonia and blood stream infections in COVID-19 patients requiring invasive mechanical ventilation: a multicenter retrospective study
Source: Ann Intensive Care. 2021 May 31;11:87. doi: 10.1186/s13613-021-00876-8 (PMC8165680; doi:10.1186/s13613-021-00876-8)
Supplement: Supplementary file 3 — Additional file 3: Table S3. Patients outcomes according to treatment with dexamethasone and rescue immunosuppressive therapy. [file 13613_2021_876_MOESM3_ESM.docx]

Table S3. Patients outcomes according to treatment with dexamethasone and rescue immunosuppressive therapy

|  | Overall  (n=151) | DEXA-/RIT- (n=47) | DEXA +/RIT-  (n=57) | DEXA -/ RIT +  (n=20) | DEXA +/RIT +  (n=27) | P value |
| --- | --- | --- | --- | --- | --- | --- |
| At least 1 VAP and/or 1 BSI, n (%) | 100 (66) | 26 (55) | 32 (56)† | 19 (95)^#^ | 23 (85)* | 0.001 |
| At least 1 VAP, n (%) | 91 (60) | 22 (47) | 31 (54) | 16 (80) | 22 (81)* | 0.005 |
| Second VAP episode, n (%) | 34 (23) | 3 (6) | 13 (23) | 9 (45)^#^ | 9 (33)* | 0.002 |
| Third VAP episode, n (%) | 15 (10) | 1 (2) | 7 (12) | 3 (15) | 4 (15) | 0.184 |
| At least 1 BSI, n (%) | 44 (29) | 8 (17) | 13 (23)† | 12 (60)^#^ | 11 (41) | 0.001 |
| Second BSI episode, n (%) | 14 (9) | 3 (6) | 2 (4) | 4 (20) | 6 (22) | 0.042 |
| Thirs BSI episode, n (%) | 3 (2) | 1 (2) | 0 | 1 (5) | 1 (4) | 0.475 |
| Mortality at D28, n (%) | 25 (17) | 9 (19) | 9 (16) | 2 (10) | 5 (19) | 0.466 |
| Mortality at D60, n (%) | 39 (26) | 11 (23) | 12 (21) | 5 (25) | 11 (41) | 0.308 |
| Hospital mortality, n (%) | 46 (32) | 12(26) | 15 (26) | 6 (30) | 13 (48) | 0.097 |
| VFD D28, median (IQR) | 0 (0-18) | 0 (0-16) | 15 (0-22)*† | 0 (0-0) | 0 (0-11)** | <0.0001 |
| VFD D60, median (IQR) | 27 (0-49) | 28 (0-48) | 45 (0-54)† | 8 (0-27) | 0 (0-43)** | 0.003 |
| Duration of mechanical ventilation, days, median (IQR) | 17 (9-37) | 18 (11-31)† | 10 (5-19)† | 43 (26-55) | 32 (11-46)** | <0.0001 |
| ICU length of stay, days, median (IQR) | 24 (15-45) | 23 (17-39)† | 16 (11-24)† | 53 (39-64) | 40 (19-50)**† | <0.0001 |
| Hospital length of stay, days, median (IQR) | 31 (20-49) | 30 (22-37)† | 24 (17-39)† | 55 (40-88) | 40 (24-50)**† | <0.0001 |
| Tukey comparisons and Bonferroni tests  *p<0.05 vs. DEXA-/RIT-  ^#^p<0.01 vs. DEXA-/RIT-  †p<0.05 vs. DEXA -/ RIT +  **p<0.05 vs. DEXA +/RIT- |  |  |  |  |  |  |
